# Effects of typhoid vaccine on inflammation and sleep in healthy participants: a double-blind, placebo-controlled, crossover study

**DOI:** 10.1007/s00213-016-4381-z

**Published:** 2016-08-09

**Authors:** Ann L. Sharpley, Charlotte M. Cooper, Clare Williams, Beata R. Godlewska, Philip J. Cowen

**Affiliations:** Neurosciences Building, Department of Psychiatry, University of Oxford, Warneford Hospital, Oxford, OX3 7JX UK

**Keywords:** Inflammation, Interleukin-6, Typhoid vaccine, Sleep continuity disturbances, Depression, Healthy participants

## Abstract

**Rationale:**

An increasing body of evidence links the occurrence of sleep continuity disturbances with increased inflammation and both sleep disturbances and inflammation are associated with clinical depression. Typhoid vaccination results in a mild inflammatory response that significantly increases levels of the proinflammatory cytokine, interleukin (IL)-6.

**Objectives:**

The present exploratory study aimed to enhance our understanding of the link between inflammation, sleep and depression by examining the effects of typhoid vaccine on the sleep polysomnogram.

**Methods:**

We studied the effects of a single injection of typhoid polysaccharide vaccine and placebo (saline solution) on sleep in 16 healthy male and female participants aged 20–38 years, sleeping at home in a randomized, double-blind, balanced order, crossover design. Subjective measures of mood, sleep and adverse effects were elicited and plasma samples analysed for IL-6 levels.

**Results:**

IL-6 levels (in picogramme per millilitre) significantly increased 2 h post vaccine compared to placebo (0.90 vs 0.53, *p* = 0.026, *r* = 0.55). Relative to placebo, typhoid vaccination produced significant impairment in several measures of sleep continuity. Total sleep time (in minute) (426.1 vs 410.7, *p* = 0.005, *r* = 0.62) and sleep efficiency percent (94.3 vs 91.5, *p* = 0.007, *r* = 0.65) were decreased; with increases in wake after sleep onset (in minute) (25.5 vs 38.8, *p* = 0.007,*r* = 0.65), total wake (in minute) (34.9 vs 50.3, *p* = 0.005,*r* = 0.67), sleep stage transitions (155.9 vs 173.1, *p* = 0.026, *r* = 0.56), number of awakenings (27.2 vs 36.1, *p* = 0.007, *r* = 0.64) and awakening index (3.8 vs 5.3, *p* = 0.005, *r* = 0.67) (means, significance level and effect size).

**Conclusions:**

Inflammatory mechanisms may underlie the impairment in sleep efficiency which is a hallmark of major depression. Because impaired sleep is also a predictor of major depression, there may be a role for suitable anti-inflammatory approaches in strategies designed to prevent the onset of depression.

ClinicalTrials.gov (http://www.clinicaltrials.gov): NCT02628054

**Electronic supplementary material:**

The online version of this article (doi:10.1007/s00213-016-4381-z) contains supplementary material, which is available to authorized users.

## Introduction

A recent systematic review and meta-analysis looking at experimental sleep deprivation studies as well as patient cohorts using a wide range of experimental designs including subjective reports, actigraphy and polysomnography (PSG) concluded that sleep disturbances and overlong sleep duration are associated with increases in markers of systemic inflammation (Irwin et al. 2015). This link appears bidirectional with poor sleep leading to elevated levels of proinflammatory cytokines, such as interleukin (IL)-6, as well as the observation that people with increased inflammatory markers due to a variety of reasons, such as rheumatoid arthritis, pregnancy, haemodialysis and increased age, experience higher levels of insomnia than in the general population (Erten et al. 2005; Friedman et al. 2005; Burgos et al. 2006; Okun et al. 2007; Fragiadaki et al. 2012). Moreover, both insomnia and chronic inflammatory illnesses are associated with high rates of co morbid depression (Dickens et al. 2002; Miller et al. 2009; Pillai et al. 2011; Felger and Lotrich 2013). Indeed symptoms of insomnia both commonly precede and co-occur with depression (Pillai et al. 2011).

Taken together, it is plausible that elevated levels of proinflammatory cytokines may be partially responsible for many of the symptoms of major depression such as low mood, insomnia and fatigue (Raison et al. 2006; Dantzer et al. 2008). Meta-analyses indicate raised levels of IL-6 in groups of patients with major depression (Dowlati et al. 2010). People with chronic disorders, such as rheumatoid arthritis exhibit raised proinflammatory cytokines and also experience higher rates of comorbid depression than would be expected in the general population. In addition, up to 50 % of hepatitis C patients become clinically depressed whilst receiving treatment with interferon-α, a recombinant form of inflammatory cytokine (Raison et al. 2005; Taylor et al. 2014). Indeed, the risk factor is such that many patients are offered prophylactic antidepressant medication before commencing interferon treatment. Such prophylactic use has good tolerability and been shown to reduce the incidence of interferon-induced depression (Udina et al. 2014).

In order to further elucidate the link between inflammation, sleep and mood we decided to use an acute inflammatory challenge in healthy participants. Previous research has shown that salmonella typhi (typhoid) vaccination stimulates a mild inflammatory response which results in increased levels of IL-6, usually without causing significant morbidity (Brydon et al. 2008; Harrison et al. 2009a). Rare side effects include anaphylaxis and serum sickness, whilst common reactions include localized swelling, discomfort and fever. The typhoid vaccination inflammation model has resulted in significant short-term lowering of mood which has been shown to correlate with increased activity within the anterior cingulate cortex during emotional face processing (Wright et al. 2005; Brydon et al. 2009; Harrison et al. 2009a).

Typhoid vaccination therefore appears a suitable means of investigating the effects of immune activation on sleep, in particular sleep continuity measures which appear linked to inflammatory responses, as well as other sleep architecture changes often observed in clinical depression (Dantzer et al. 2008; Pillai et al. 2011; Motivala et al. 2005).

Studying healthy participants enables investigation of the effects of inflammatory challenge in individuals who do not currently have an inflammatory condition, or alterations to their sleep patterns due to a sleep disorder or as part of a depressive condition. We therefore assessed the effect of typhoid vaccination on the PSG in healthy subjects using home-based ambulatory recording, previously shown to be sensitive in detecting the effects of psychotropic drugs on the PSG (Sharpley et al. 1994, 2011).

## Methods and materials

### Participants and study design

We studied 16 healthy participants (9 female, 7 male; mean age 26.6 years; range 20–38 years; BMI 23; range 18.8–26.4). Eleven of the participants had not received the typhoid vaccine before. Participants were recruited through local and online advertising. Inclusion criteria included: participant willing and able to give informed consent, not currently taking any medications (except the contraceptive pill) and a good sleeper determined by self-report and a sleep disorders screening interview. Exclusion criteria included: any current or previous DSM-IV Axis 1 psychiatric disorder (determined using the Standard Clinical Interview for Diagnostic and Statistical Manual for Mental Health Disorders- Fourth Edition) (Spitzer et al. 1995), diagnosis of current sleep disorder, any significant current medical condition likely to interfere with the conduct of the study or analysis of data, typhoid vaccination within the last 3 years, any vaccination within the last 6 months, history of allergies to drugs or vaccines or any component of the typhoid vaccine, congenital or acquired immune deficiency, bleeding disorder, current or recent physical illness or infection within previous 2 weeks, steroidal or non-steroidal anti-inflammatory medication within preceding 2 weeks, current substance misuse, child bearing age and not using reliable form of contraception, pregnant or breast feeding, and finally, has taken part in a psychological or medical experiment involving taking any kinds of drugs within the last 6 weeks.

All participants signed and dated the Informed Consent Form before any study specific procedures were performed. During the screening visit, in order to characterize the sample, participants completed the Eysenck Personality Questionnaire (EPQ) (Eysenck and Eysenck 1975), State Trait Anxiety Inventory (STAI) (Spielberger et al. 1970) and Beck Depression Inventory (BDI-II) (Beck et al. 1996) (Table 1). For the two study visits, participants attended the department between 1445 and 1730 hours. Following a brief interview to ensure that there had been no changes in health or medication since the screening visit that could affect eligibility, each participant was given a single 0.5-mL injection of either typhoid polysaccharide vaccine (Typhim Vi®) (Sanofi Pasteur MSD Limited, Berkshire, UK) or placebo (0.9 % sodium chloride saline solution) into the non-dominant deltoid muscle in the arm in a randomized, double blind, balanced order, cross-over design with a 7 to 14 day washout period between each study visit. Each 0.5-mL dose of typhoid vaccine contains 25 μg of the active substance, purified Vi capsular polysaccharide of *Salmonella typhi* (Ty2 strain). The other ingredients are phenol, sodium chloride, disodium phosphate dihydrate, sodium dihydrogen phosphate dihydrate and water. This vaccine is generally well-tolerated, but some individuals can experience temporary soreness, swelling, redness or hardness at the site of the injection. Less common side effects can include pyrexia, headache, nausea, diarrhoea, generally feeling unwell, abdominal pain and asthma with severe reactions being rare. All participants were closely monitored for 2 h post injection and a researcher was available by phone throughout the study.

**Table 1:** Effect of typhoid vaccine and placebo, on selected sleep parameters (*n* = 15)

Selected sleep parameters ^a^	Group mean	SE	*t* (*df* = 14)	Two-tailed significance, *p* value	*r* (effect size)
Sleep continuity measures					
Total sleep time					
Placebo	426.1	13.0	3.354	0.005	0.62
Typhoid vaccine	410.7	12.2			
Sleep efficiency, % ^b^					
Placebo	94.3	0.8	3.171	0.007	0.65
Typhoid vaccine	91.5	1.0			
Wake after sleep onset					
Placebo	25.5	3.5	−3.183	0.007	0.65
Typhoid vaccine	38.8	4.8			
Total wake					
Placebo	34.9	3.4	−3.365	0.005	0.67
Typhoid vaccine	50.3	5.0			
Sleep stage transitions					
Placebo	155.9	8.0			
Typhoid vaccine	173.1	9.2	−2.5	0.026	0.56
No. of awakenings					
Placebo	27.2	1.9	−3.1	0.007	0.64
Typhoid vaccine	36.1	3.5			
Awakening index					
Placebo	3.8	0.3			
Typhoid vaccine	5.3	0.5	−3.337	0.005	0.67
Sleep onset latency					
Placebo	9.4	1.2	−0.851	0.4	0.22
Typhoid vaccine	11.6	2.6			
NREM sleep measures					
N 1 (stage 1)					
Placebo	37.8	2.8	−1.409	0.2	0.35
Typhoid vaccine	45.0	5.2			
N 2 (stage 2)					
Placebo	140.7	8.8	1.167	0.3	0.30
Typhoid vaccine	133.0	8.2			
N3 (SWS)					
Placebo	140.8	6.8	1.588	0.1	0.30
Typhoid vaccine	128.7	6.9			
REM sleep measures					
REM latency					
Placebo	65.6	4.9	−0.677	0.5	0.18
Typhoid vaccine	69.5	4.7			
REM sleep					
Placebo	106.8	6.8	0.739	0.4	0.20
Typhoid vaccine	103.6	5.4			

^a^Sleep parameters expressed in minutes unless stated otherwise

^b^Sleep efficiency % = actual sleep time/time in bed × 100

Abbreviations: *NREM* non-rapid eye movement, *REM* rapid eye movement, *SWS* slow wave sleep

### Subjective questionnaires

Participants completed a set of subjective measures of mood state, hourly, for 4 h post injection. The Positive and Negative Affect Schedule (PANAS) is a psychometric scale, with ten descriptives for each positive and negative affect rated on a five-point Likert Scale ranging from ‘very slightly’ to ‘very much’ (Watson et al. 1988). Participants were asked to rate how they feel *right now* and two scores obtained; positive and negative (range 10–50). The Bond and Lader visual analogue scales (VAS) consisting of sixteen 100-mm VAS anchored by antonyms (e.g. alert–drowsy, lethargic-energetic, etc.), were combined as recommended by the authors to form three mood factors: alertness, calmness and contentedness with higher scores reflecting higher subjective feelings of stress (Bond and Lader 1974). An adverse effects profile looked at the presence and severity of common adverse effects; nausea, vomiting, dizziness, headache, loss of alertness, drowsiness and increased and decreased appetite. In addition, participants were asked to report any other symptoms that they noted.

### IL-6 immunoassay

Five milliliter blood samples were collected between February 16, 2015 and July 6, 2015 into an EDTA tube 2 h post injection, centrifuged for 10 min at 1,250×*g* within 30 min. Plasma was then stored at −30 °C until assay on September 23, 2015. On study completion, the plasma samples were transferred in a single batch, in unlinked anonymised form, for Human IL-6 Immunoassay using a Quantikine® HS (high sensitivity) ELISA kit (R&D Systems, Abingdon, UK). The intra-assay and inter-assay coefficients of variation are 7.4 and 7.8 %, respectively, and the limit of detection of the assay is 0.039 pg/mL.

### Polysomnograms

The electrodes were fitted during each study visit and the participants then returned home to sleep as usual. On each study night, PSGs were recorded using the Embla Titanium recording system (Natus Neurology Incorporated, Middleton, WI 53562 USA). Participants retired and rose at their usual time, and this was kept constant for both study nights and preceding nights. We have previously demonstrated that the use of ambulatory home sleep recordings provides a reliable and valid method for PSG data collection and does not result in first night effects often seen in sleep laboratories (Sharpley et al. 1988, 1990). The industry standard montage, American Academy of Sleep Medicine (AASM), was used which comprises six electroencephalogram (EEG) channels (C3-M2, C4-M1, O1-M2, O2-M1, F3-M2, F4-M1), two electrooculogram (EOG) channels (E1-M2 and E2-M2), and submentalis electromyogram (EMG) channels using three electrodes (Iber et al. 2007). PSGs were staged in 30-s epochs using the Embla RemLogic sleep diagnostic software (Natus Neurology Incorporated, Middleton, WI, USA). RemLogic adheres to the AASM scoring criteria (Iber et al. 2007). AASM criteria have reclassified sleep stages and slow wave sleep (SWS) is now known as N3. Additionally, the PSGs were edited by one of the authors, an experienced sleep physiologist who was blind to treatment status (ALS). Participants were instructed not to consume alcohol, high-fat-content meals and to avoid intensive exercise for 24 h prior to each study visit. Normal caffeine intake was maintained to ensure caffeine withdrawal was not an issue. The following morning, participants removed the electrodes themselves and, within 20 min of waking, participants completed the 10-item Leeds Sleep Evaluation Questionnaire (LSEQ) (Parrott and Hindmarch, 1980). This is a standardized VAS looking at four aspects of sleep: Getting to Sleep (GTS), Quality of Sleep (QOS), Awake following Sleep (AFS) and Behaviour following Awakening (BFW) following a medication compared to the participants’ usual sleep. In addition, a final Adverse Effects profile was completed and participants were also asked to guess, with degree of certainty, whether they had received typhoid vaccine or placebo.

### Study oversight

The first author vouches for the accuracy and completeness of the data and for the fidelity of the study to the protocol. Concealment of randomization was maintained throughout the entire study for all participants and for all researchers involved in data acquisition and analysis and the code broken only after all data had been analysed. The randomization schedule was drawn up by a member of the group not otherwise involved in the study to maintain allocation concealment.

### Statistical analysis

Sixteen participants were recruited for this study. Based on previous PSG studies, this has a statistical power of 0.8 to detect a 5 % change in % sleep efficiency (SE) with <0.05 threshold. Statistical analyses were performed in SPSS version 22 (www.ibm.com/software/analytics/spss). Differences between pairs of sleep nights, LSEQ and IL-6 levels were assessed using Dependent-means (paired-samples) *t* test (two tailed) and effect sizes (*r*) calculated. Individual mood and side effect questionnaire data were analysed using a repeated measures ANOVA with two within subject factors, ‘treatment’ (placebo versus typhoid vaccine) and ‘time’ (time since treatment administration).

## Results

The typhoid vaccine was well tolerated and no participant dropped out of the study. One participant was removed from the PSG analysis due to unusual and disturbing noise outside his house preventing him sleeping on one night. His subjective pre-sleep data have been included in the analyses. Due to difficulty obtaining the sample, one participant did not provide a blood sample.

### Demographics

The baseline questionnaires (BDI-II, STAI and EPQ) showed that the participants’ data were in the normal range [Electronic supplementary material (ESM) Table S1].

### PSGs

**Fig. 1:**
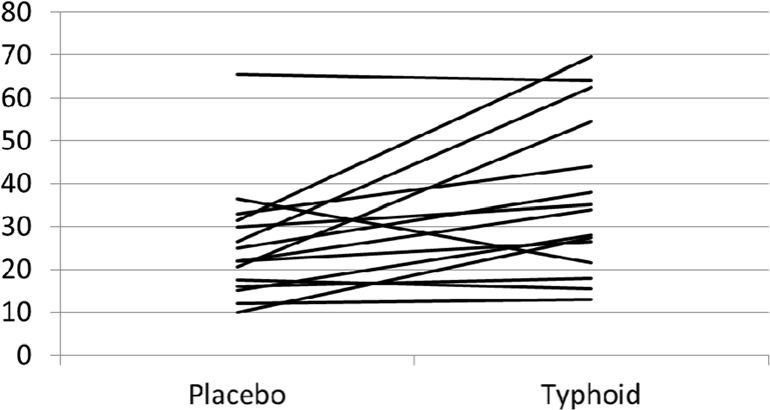
Effect of typhoid vaccine and placebo on wake after sleep onset (minutes) *n* = 15. Paired samples *t* test mean = *p* = 0.007

Typhoid vaccine produced several changes in sleep continuity parameters using Dependent means (paired samples) *t* test (two tailed) (Table 1). Specifically, total sleep time (TST) (in minute) (*p* = 0.005, *r* = 0.62) and SE% (*p* = 0.007, *r* = 0.65) were significantly decreased; consistent with this, there were significant increases in wake after sleep onset (WASO) (in minute) (*p* = 0.007, *r* = 0.65) (Fig. 1), total wake (in minute) (*p* = 0.005, *r* = 0.67), sleep stage transitions (*p* = 0.026, *r* = 0.56), number of awakenings (*p* = 0.007, *r* = 0.64) and awakening index (*p* = 0.005, *r* = 0.67) following typhoid vaccine compared to the saline injection. No other sleep parameters were significantly altered (all *p* > 0.05).

### Inflammatory marker

**Fig. 2:**
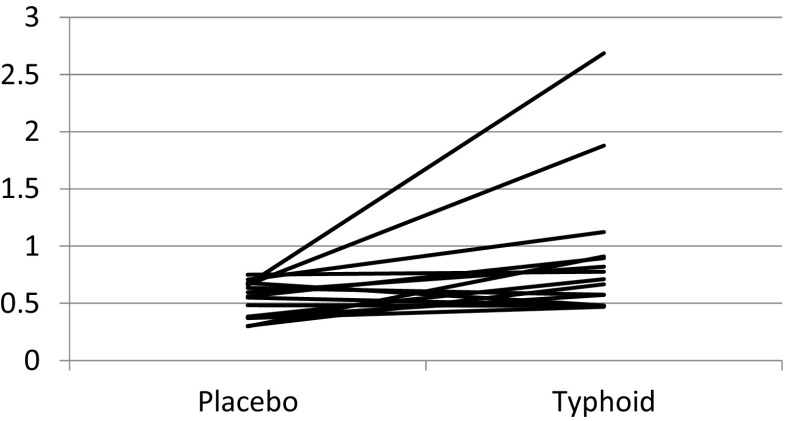
Effect of typhoid vaccine and placebo on plasma IL-6 (in picogramme per millilitre) levels. *n* = 15 paired *t* test *P* = 0.026

IL-6 levels (in picogramme per millilitre) were significantly increased 2 h post typhoid vaccine (0.90 ± 0.16) compared to the saline injection (0.53 ± 0.04), (mean ± SE), t(14) = −2.48, *P* = 0.026, *r* = 0.55 (Fig. 2). Despite the increase, no significant correlations were found between changes in IL-6 levels 2 h post vaccine and any of the significant sleep continuity measures.

### Subjective questionnaires

All subjective-state measures were unaffected by typhoid administration: PANAS,

Bond and Lader VAS and LSEQ (all *p* values >0.1; ESM Tables S2 and S3). The Adverse effects profile looked at both presence and severity of symptoms. The effects were mild and similar for both typhoid vaccine and placebo condition with the exception of ‘sore shoulder’, with a significant main effect of treatment (*F* = 6.5, *df* (1, 15), *p* = 0.023) but no treatment by time interaction (*F* = 3.6 *df* (1, 4), *p* = 0.063). As expected, ratings of sore shoulder were greater after typhoid vaccination than placebo injection. (Fig. Fig. S1). The participants’ randomization guess revealed that participants could guess, despite mild adverse effects, the difference between the typhoid vaccine and placebo with 13/16 guessing correctly on both nights but with more certainty on the placebo (62.6 %) compared to typhoid vaccine (55.2 %) occasion.

## Discussion

Previous studies in our laboratory have shown that single doses of psychotropic drugs can cause acute changes in sleep architecture and continuity (Sharpley et al. 1994, 2011) Typhoid vaccine has been shown in several healthy volunteer studies to induce short-term lowering of mood and we have therefore employed this model to explore the link between inflammation and sleep markers in depression. (Wright et al. 2005; Brydon et al. 2009; Harrison et al. 2009b).

Our findings suggest that several measures of sleep continuity, including significant decreases in TST and SE% and significant increases in WASO, total wake, sleep stage transitions, number of awakenings and awakening index, are disrupted following typhoid vaccine compared to placebo. In our study, typhoid vaccine elicited a significant increase in levels of IL-6 2 h post-dose demonstrating a small but significant acute inflammatory activation, and suggests the start of the response that is usually driven by IL-6 and is most likely responsible for the disruptions to sleep continuity. The magnitude of the increase in IL-6 levels observed in our study are similar to those found in previous research which sampled at 2 h (Brydon et al. 2009), and a 24-h time course shows that peak IL-6 levels occur 6–7 h post injection, returning to baseline levels after 24 h (Paine et al. 2013). It should also be noted that in depression there does not appear to be a significant correlation between IL-6 levels in blood and those in CSF (Sasayama et al. 2013).

However, correlations between IL-6 and sleep continuity measures were not significant**.** One possible explanation is that for procedural reasons, blood was taken 2 h post vaccination, thus the participants IL-6 levels at bedtime are unknown and it is thus not possible to determine whether correlations between IL-6 levels and measures of sleep continuity would have been significant if taken at a later time point. Moreover, given by itself, IL-6 appears to have the ability to increase SWS and SE (Benedict et al. 2009). Therefore, it seems likely that some other inflammatory mediator is responsible for the sleep disruption caused by typhoid vaccination. Krueger et al. (2008) have pointed out that the mechanisms by which cytokines modify sleep probably involves action on local neural networks with IL-1, for example, enhancing inhibition of hypothalamic glutamatergic neurons. Sleep disruption could be a consequence of altered activity in these local networks which prevents the emergence of sleep from co-ordinated neuronal assemblies.

It has been documented that sleep disturbance has an adverse influence on the risk of infectious and inflammatory disease, the incidence of depression and the occurrence and progression of illnesses such as cancer and cardiovascular disease (Dew et al. 2003; Vgontzas et al. 2013; Irwin et al. 2015). Our current research, using typhoid vaccine as an experimental model, increases our knowledge of the reciprocal links between inflammation and sleep impairment.

Meta-analyses have shown that during an episode of depression, rapid eye movement (REM) sleep and density are increased and REM latency, SWS, TST and SE% are decreased compared to matched controls (Erten et al. 2005). In our present study, we did not see the characteristic changes in SWS and REM sleep associated with depression; however, impaired sleep continuity, which both predicts and varies with the clinical state of depression, was apparent. This suggests that although inflammation could play a role in the sleep continuity changes that occur in depression, it does not account for the changes in sleep architecture. However, it is possible that sleep architecture changes might become apparent if a stronger inflammatory stimulus was used or if the inflammatory state persisted for a longer time. A recent systematic review and meta-analysis looking at both cohort and experimental sleep deprivation studies concluded that sleep disturbance is associated with increases in markers of systemic inflammation (Irwin et al. 2015).

Although the majority of participants correctly guessed the order they had been given the typhoid vaccine and placebo injection, few adverse effects were reported and only the incidence of a sore shoulder was significantly different between the two study conditions, with no participants reporting an awareness of a sore shoulderfollowing saline injection whereas at 4 h post typhoid vaccination 3/16 reported soreness and by the following morning this had increased to 5/16 compared to 0/16 for the placebo session. However, this accounts for a minority of participants and all reported this symptom as mild or moderate. Therefore although we cannot exclude this symptom as a possible factor to explain the impaired sleep on the typhoid vaccine night, it is unlikely.

A further limitation was that unlike previous studies (Wright et al. 2005; Brydon et al. 2009; Harrison et al. 2009b), we were unable to detect acute changes in subjective mood**.** It is possible that the mood questionnaires employed in our healthy, young, mainly student population were not sensitive enough to detect changes between conditions. Thus whilst typhoid vaccination may be a good model of a mild inflammatory state, it does not reliably induce depression and therefore may not be a good way to model an inflammatory-depressive condition. A discrepancy between subjective sleep, using the LSEQ, and objective sleep, with PSG, was also apparent. An average increase in wake time of 15 min spread over the entire night on the typhoid vaccine session may not be sufficient to elicit a worsening of subjective sleep when sleep efficiency is already high in these good sleepers.

In conclusion, our findings indicate that an acute, mild inflammatory stimulus produces impairments in sleep continuity resulting in decreased sleep efficiency. Decreased sleep efficiency is a hallmark of depression and it is possible that inflammatory mechanisms play a role in this disturbance. In addition, impaired sleep is a precursor of depression and an independent predictor of suicidality (Wojnar et al. 2009). In people suffering from insomnia linked to inflammatory processes the use of suitable anti-inflammatory strategies could improve sleep as well as perhaps preventing the onset of depression and decreasing the risk of suicidality.

## Electronic supplementary material

Fig. S1 (DOCX 83 kb)

Table S1 (DOCX 15 kb)

Table S2 (DOCX 16 kb)

Table S3 (DOCX 16 kb)

## References

[CR1] Beck AT, Steer RA, Brown GK (1996). Manual for the Beck depression inventory–II.

[CR2] Benedict C, Scheller J, Rose-John S, Born J, Marshall (2009). Enhancing influence of intranasal interleukin-6 on slow wave activity and memory consolidation during sleep. FASEB J.

[CR3] Bond A, Lader M (1974). The use of analogue scales in rating subjective feelings. Br J Med Psychol.

[CR4] Brydon L, Harrison NA, Walker C, Steptoe A, Critchley HD (2008). Peripheral inflammation is associated with altered substantia nigra activity and psychomotor slowing in humans. Biol Psychiatry.

[CR5] Brydon L, Walker C, Wawrzyniak A, Whitehead D, Okamura H, Yajima (2009). Synergistic effects of psychological and immune stressors on inflammatory cytokine and sickness responses in humans. Brain Behav Immun.

[CR6] Burgos I, Richter L, Klein T, Fiebich B, Feige B, Lieb K (2006). Increased nocturnal interleukin-6 excretion in patients with primary insomnia: a pilot study. Brain Behav Immun.

[CR7] Dantzer R, O’Connor JC, Freund GG, Johnson RW, Kelley KW (2008). From inflammation to sickness and depression: when the immune system subjugates the brain. Nat Rev Neurosci.

[CR8] Dew MA, Hoch CC, Buysse DJ, Monk TH, Begely AE, Houck PR (2003). Healthy older adults’ sleep predicts all-cause mortality at 4 to 19 years of follow-up. Psychosom Med.

[CR9] Dickens C, Mcgowan L, Clark-Carter D, Creed F (2002). Depression in rheumatoid arthritis: a systematic review of the literature with meta-analysis. Psychosom Med.

[CR10] Dowlati Y, Herrmann N, Swardfager W, Liu H, Sham L, Reim EK, Lanctôt KL (2010). A meta-analysis of cytokines in major depression. Biol Psychiatry.

[CR11] Erten Y, Kokturk O, Yuksel A, Elbeg S, Ciftci TU, Pasaoglu H (2005). Relationship between sleep complaints and pro inflammatory cytokines in haemodialysis patients. Nephrology.

[CR12] Eysenck HJ and Eysenck SBG (1975): In: Manual of the Eysenck Personality Questionnaire (adult and junior). London: Hodder & Stoughton

[CR13] Felger JC, Lotrich FE (2013). Inflammatory cytokines in depression: neurobiological mechanisms and therapeutic implications. Neuroscience.

[CR14] Fragiadaki K, Tektonidou MG, Konsta M, Chrousos GP, Sfikakis PP (2012). Sleep disturbances and interleukin 6 receptor inhibition in rheumatoid arthritis. J Rheumatol.

[CR15] Friedman EM, Hayney MS, Love GD, Urry HL, Rosenkranz MA, Davidson RJ (2005). Social relationships, sleep quality, and interleukin-6 in aging women. Proc Natl Acad Sci U S A.

[CR16] Harrison NA, Brydon L, Walker C, Gray MA, Steptoe A, Dolan RJ (2009). Neural origins of human sickness in interoceptive responses to inflammation. Biol Psychiatry.

[CR17] Harrison NA, Brydon L, Walker C, Gray MA, Steptoe A, Critchley HD (2009). Inflammation causes mood changes through alterations in subgenual cingulate activity and mesolimbic connectivity. Biol Psychiatry.

[CR18] Iber C, Ancoli-Israel S, Chesson A, Quan S, for the American Academy of Sleep Medicine (2007). The AASM manual for the scoring of sleep and associated events: rules, terminology and technical specifications.

[CR19] Irwin MR, Olmstead R, Carroll JE (2015). Sleep disturbance, sleep duration, and inflammation: a systematic review and meta-analysis of cohort studies and experimental sleep deprivation. Biol Psychiatry.

[CR20] Krueger JM (2008) The role of cytokines in sleep regulation. Curr Pharm Des. 14:3408–16 10.2174/138161208786549281 PMC2692603 19075717

[CR21] Miller AH, Maletic V, Raison CL (2009). Inflammation and its discontents: the role of cytokines in the pathophysiology of major depression. Biol Psychiatry.

[CR22] Motivala SJ, Sarfatti A, Olmos L, Irwin MR (2005). Inflammatory markers and sleep disturbance in major depression. Psychosom Med.

[CR23] Okun ML, Hall M, Coussons-Read ME (2007). Sleep disturbances increase interleukin-6 production during pregnancy: implications for pregnancy complications. Reprod Sc.

[CR24] Parrott AC, Hindmarch I (1980). The Leeds sleep evaluation questionnaire in psychopharmacological investigations—a review. Psychopharmacology.

[CR25] Paine NJ, Ring C, Bosch JA, Drayson MT, Veldhuijzen van Zanten JJ (2013). The time course of the inflammatory response to the salmonella typhi vaccination. Brain Behav Immun.

[CR26] Pillai V, Kalmbach DA, Ciesla JA (2011). A meta-analysis of electroencephalographic sleep in depression: evidence for genetic biomarkers. Biol Psychiatry.

[CR27] Raison CL, Borisov AS, Broadwell SD, Capuron L, Woolwine BJ, Jacobson IM (2005). Depression during pegylated interferon-alpha plus ribavirin therapy: prevalence and prediction. J Clin Psychiatry.

[CR28] Raison CL, Capuron L, Miller AH (2006). Cytokines sing the blues: inflammation and the pathogenesis of depression. Trends Immunol.

[CR29] Sasayama D, Hattori K, Wakabayashi C, Teraishi T, Hori H, Ota M (2013). Increased cerebrospinal fluid interleukin-6 levels in patients with schizophrenia and those with major depressive disorder. J Psychiatr Res.

[CR30] Sharpley AL, Elliott JM, Attenburrow MEJ, Cowen PJ (1994). Slow wave sleep in humans: role of 5-HT_2A_ and 5-HT_2C_ receptors. Neuropharmacology.

[CR31] Sharpley AL, Solomon RA, Cowen PJ (1988). Evaluation of first night effect using ambulatory monitoring and automatic sleep stage analysis. Sleep.

[CR32] Sharpley AL, Solomon RA, Cowen PJ (1990). Sleep stability with home sleep recording and automatic sleep stage analysis. Sleep.

[CR33] Sharpley AL, Rawlings NB, Brain S, McTavish SFB, Cowen PJ (2011). Does agomelatine block 5HT2C receptors in humans?. Psychopharmacology.

[CR34] Spielberger CD, Gorsuch RL, Lushene RE (1970) Manual for the State-Trait Anxiety Inventory. at http://ubir.buffalo.edu/xmlui/handle/10477/2895

[CR35] Spitzer RL, Williams JBW, Gibbon M (1995). Structured clinical interview for DSM-IV (SCID).

[CR36] Taylor MJ, Godlewska B, Near J, Christmas D, Potokar J, Collier J (2014). Effect of interferon-α on cortical glutamate in patients with hepatitis C: a proton magnetic resonance spectroscopy study. Psychol Med.

[CR37] Udina M, Hidalgo D, Navinés R, Forns X, Solà R, Farré M, Capuron L, Vieta E, Martín-Santos R (2014). Prophylactic antidepressant treatment of interferon-induced depression in chronic hepatitis C: a systematic review and meta-analysis. J Clin Psychiatry.

[CR38] Vgontzas AN, Fernandez-Mendoza J, Liao D, Bixler EO (2013). Insomnia with objective short sleep duration: the most biologically severe phenotype of the disorder. Sleep Med Rev.

[CR39] Watson D, Clark LA, Tellegen (1988). A development and validation of brief measures of positive and negative affect: the PANAS scales. J Pers Soc Psychol.

[CR40] Wojnar M, Ilgen MA, Wojnar J, McCammon RJ, Valenstein M, Brower KJ (2009). Sleep problems and suicidality in the National Comorbidity Survey Replication. J Psychiatric Research.

[CR41] Wright C, Strike P, Brydon L, Steptoe A (2005). Acute inflammation and negative mood: mediation by cytokine activation. Brain Behav Immun.

