# Supplementary material for: Effects of typhoid vaccine on inflammation and sleep in healthy participants: a double-blind, placebo-controlled, crossover study
Source: Psychopharmacology (Berl). 2016 Aug 9;233:3429–35. doi: 10.1007/s00213-016-4381-z (PMC4989013; doi:10.1007/s00213-016-4381-z)
Supplement: Supplementary file 1 — (DOCX 83 kb) [file 213_2016_4381_MOESM1_ESM.docx]

**Figure S1.** Effect of Typhoid Vaccine and Placebo on the incidence and severity of Adverse Effects taken hourly for 4 hours post injection and the following morning. n=16
