# Supplementary material for: Effects of typhoid vaccine on inflammation and sleep in healthy participants: a double-blind, placebo-controlled, crossover study
Source: Psychopharmacology (Berl). 2016 Aug 9;233:3429–35. doi: 10.1007/s00213-016-4381-z (PMC4989013; doi:10.1007/s00213-016-4381-z)
Supplement: Supplementary file 2 — (DOCX 15 kb) [file 213_2016_4381_MOESM2_ESM.docx]

**Table S1.** Baseline Mood and Personality Questionnaires.

Means (standard deviations). n=16

| **Parameter** | **Mean Score** | **SD** | **Questionnaire**  **Range** |
| --- | --- | --- | --- |
| STAI State anxiety | 26.5 | 5.1 | 20-80 |
| BDI-II | 1.2 | 2.2 | 0-63 |
| EPQ: E | 13.2 | 5.1 | 0-23 |
| EPQ: N | 6.9 | 3.0 | 0-24 |
| EPQ: P | 2.2 | 2.4 | 0-32 |
| EPQ: L | 7.1 | 2.7 | 0-21 |

STAI: State Trait Anxiety Index; BDI: Beck Depression Inventory; EPQ: Eysenck Personality Questionnaire, where E: extroversion, N: neuroticism, P: psychoticism, L: lie.
