# Supplementary material for: Effects of typhoid vaccine on inflammation and sleep in healthy participants: a double-blind, placebo-controlled, crossover study
Source: Psychopharmacology (Berl). 2016 Aug 9;233:3429–35. doi: 10.1007/s00213-016-4381-z (PMC4989013; doi:10.1007/s00213-016-4381-z)
Supplement: Supplementary file 3 — (DOCX 16 kb) [file 213_2016_4381_MOESM3_ESM.docx]

**Table S2.** Effect of Typhoid Vaccine and Placebo, on Subjective Mood ratings using the Positive and Negative Affective Schedule (PANAS) questionnaire and Bond and Lader VAS. Means (standard deviations). n=16

|  | **Placebo** |  |  |  | **Typhoid** |  |  |  |  |
| --- | --- | --- | --- | --- | --- | --- | --- | --- | --- |
|  | **1h** | **2h** | **3h** | **4h** | **1h** | **2h** | **3h** | **4h** | **P value** |
| **PANAS** |  |  |  |  |  |  |  |  |  |
| Positive | 30.0±6.2 | 28.4±6.2 | 27.9±6.1 | 27.9±5.6 | 30.2±6.3 | 29.1±6.5 | 25.9±6.9 | 26.5±8.2 | .8 |
| Negative | 12.0±3.2 | 11.9±2.6 | 10.9±1.7 | 10.9±1.8 | 11.2±1.9 | 10.6±1.1 | 10.9±1.6 | 10.9±1.8 | .4 |
| **Bond- Lader Mood Factors** |  |  |  |  |  |  |  |  |  |
| Alertness | 47.9±4.4 | 47.7±3.0 | 47.0±4.4 | 48.0±4.5 | 46.5±3.6 | 46.9±4.6 | 48.5±3.1 | 50.3±3.0 | .7 |
| Calmness | 50.5±5.6 | 50.8±4.3 | 52.5±4.5 | 52.6±4.6 | 50.1±5.5 | 51.9±4.1 | 53.3±4.9 | 51.2±5.3 | 1.0 |
| Contentedness | 52.0±7.0 | 49.8±8.1 | 49.5±11.7 | 54.8±7.6 | 52.7±8.2 | 50.6±8.5 | 52.2±9.2 | 53.9±8.5 | .7 |
