# Supplementary material for: Effects of typhoid vaccine on inflammation and sleep in healthy participants: a double-blind, placebo-controlled, crossover study
Source: Psychopharmacology (Berl). 2016 Aug 9;233:3429–35. doi: 10.1007/s00213-016-4381-z (PMC4989013; doi:10.1007/s00213-016-4381-z)
Supplement: Supplementary file 4 — (DOCX 16 kb) [file 213_2016_4381_MOESM4_ESM.docx]

**Table S3.** Effect of Typhoid Vaccine and Placebo, on the Leeds Sleep Evaluation Questionnaire (LSEQ) (n=15)

| **Subjective Sleep Measures** | **Group Mean + SD** | **Difference of Means (SD)** | **95 % CI** | **t** | **2-Tailed Significance,**  **p Value** |
| --- | --- | --- | --- | --- | --- |
| **GTS** |  |  |  |  |  |
| Placebo | 47.7 ±7.0 | -1.3 (12.6) | -8.0 to 5.4 | -.415 | .7 |
| Typhoid Vaccine | 49.0±11.4 |  |  |  |  |
| **QOS** |  |  |  |  |  |
| Placebo | 43.3±11.1 | -2.0 (9.4) | -7.0 to 3.0 | -.847 | .4 |
| Typhoid Vaccine  **AFS**  Placebo  Typhoid Vaccine  **BFW**  Placebo  Typhoid Vaccine | 45.3±9.2    47.5±9.9  51.0±11.4    53.4±14.6  53.4±12.2 | -3.4(15.5)  0.02(10.6) | -11.7 to 4.8  -5.6 to 5.7 | -.886  .008 | .4  .9 |
|  |  |  |  |  |  |

GTS=Getting to Sleep; QOS=Quality of Sleep; AFS=Awake following Sleep;

BFW= Behaviour following wakening
